# Differential Blood–Brain Barrier Transport and Cell Uptake of Cyclic Peptides In Vivo and In Vitro

**DOI:** 10.3390/pharmaceutics15051507

**Published:** 2023-05-16

**Authors:** Erik Melander, Camilla Eriksson, Sara Wellens, Kimia Hosseini, Robert Fredriksson, Fabien Gosselet, Maxime Culot, Ulf Göransson, Margareta Hammarlund-Udenaes, Irena Loryan

**Affiliations:** 1 Department of Pharmacy, Uppsala University, 75123 Uppsala, Sweden; erik.melander@farmaci.uu.se (E.M.); margareta.hammarlund-udenaes@farmaci.uu.se (M.H.-U.); 2 Department of Pharmaceutical Biosciences, Uppsala University, 75123 Uppsala, Sweden; camilla.eriksson@fkog.uu.se (C.E.); kimia.hosseini@farmbio.uu.se (K.H.); robert.fredriksson@farmbio.uu.se (R.F.); ulf.goransson@farmbio.uu.se (U.G.); 3 Laboratoire de la Barrière Hémato-Encéphalique (LBHE), Faculté des Sciences Jean Perrin, University of Artois, UR 2465, Rue Jean Souvraz SP18, F-62300 Lens, Francefabien.gosselet@univ-artois.fr (F.G.); maxime_culot@icloud.com (M.C.)

**Keywords:** cyclic peptide, cell-penetrating peptide, kalata B1, SFTI-1, blood–brain barrier, intracellular distribution, pharmacokinetics, CNS scaffold

## Abstract

The blood–brain barrier (BBB) poses major challenges to drug delivery to the CNS. SFTI-1 and kalata B1 are cyclic cell-penetrating peptides (cCPPs) with high potential to be used as scaffolds for drug delivery. We here studied their transport across the BBB and distribution within the brain to gauge the potential of these two cCPPs as scaffolds for CNS drugs. In a rat model, SFTI-1 exhibited, for a peptide, high extent of BBB transport with a partitioning of unbound SFTI-1 across the BBB, K_p,uu,brain_, of 13%, while only 0.5% of kalata B1 equilibrated across the BBB. By contrast, kalata B1, but not SFTI-1, readily entered neural cells. SFTI-1, but not kalata B1, could be a potential CNS delivery scaffold for drugs directed to extracellular targets. These findings indicate that differences between the BBB transport and cellular uptake abilities of CPPs are crucial in the development of peptide scaffolds.

## 1. Introduction

The blood–brain barrier (BBB) is a dynamic bidirectional defence interface that protects the brain from potentially harmful endogenous and exogenous solutes present in the systemic circulation and in the brain interstitial fluid (ISF). It is also a formidable barrier to many conventional small molecular weight drugs as well as large-molecule therapeutics, such as peptide and protein drugs. Attempts have been made to use different invasive and non-invasive technologies to improve drug delivery across the BBB [1]. Among biological mechanisms based on non-invasive technologies, enhancement of central nervous system (CNS) drug delivery via linear or cyclic cell-penetrating peptides (cCPPs) and their scaffolds has its unique place [2,3,4]. In spite of high promise, there is currently no CPP or CPP/cargo complex approved for clinical use for the treatment of CNS diseases or CNS delivery [2,5]. The latter is primarily attributed to translational issues on various levels, including in vitro–in vivo extrapolation.

The BBB comprises capillary endothelial cells sharing their basal lamina with pericytes and surrounded by astrocytes and other extracellular matrix components, microglia, and neurons. Together they orchestrate the regulation of the highly specialized functions of the brain endothelium, such as minimal pinocytotic activity, very low rate of transcytosis, and selective transport into and out of the brain [6,7]. Large molecules including peptides are generally considered to be excluded from the brain; however, some may be internalized by receptor or absorptive-mediated uptake at the BBB and further sorted via the vesicular transport machinery [8,9] and then recycled back to the cell surface, degraded, or transcytosed to the opposite side of the endothelial cell [9]. The ability of cCPPs to engage targets with high affinity and specificity, tunability, and ease of synthesis make them the most attractive modalities in modern drug discovery, despite the difficulties in crossing the BBB. 

CPPs and cCPPs are suggested to be good drug-delivery agents [10,11,12,13]; yet, to our knowledge, there are no in vivo studies focusing on the quantitate characterization of unbound-peptide pharmacokinetics for validation of their CNS drug delivery. Whilst many peptides have been evaluated as BBB shuttles, these are primarily linear peptides. In spite of years of investigating CPPs, their transport ability across the BBB and specific mechanisms related to the probable vesicular trafficking across brain endothelial cells remain unknown. cCPPs are typically 5–30 amino acids in length and have a protein-derived, chimeric, and synthetic nature [2,14]. They have garnered much interest because of their potential use for intracellular delivery of otherwise membrane-impermeable therapeutics. Compared with linear peptides, cCPPs have several reported advantages, such as high resistance to proteolysis, thermostability, increased cell permeability, efficient endosomal escape, and improved ability to conjugate (i.e., ease of modification) with therapeutic and imaging agents [14,15,16]. However, while their potential for use as carriers of drugs into the brain could be high, pertinent proof-of-concept studies come from in vitro settings [17,18,19], missing validation from in vivo brain pharmacokinetic studies.

Disulphide bond cyclic peptides are an intriguing and promising class of molecules that, in principle, could be used as scaffolds for drug delivery for treatment of CNS disorders. If their purported cell-penetrating properties are present in brain endothelial cells, it would widen the perspectives to study cCPPs in the context of brain drug delivery. Two notable cCPPs are sunflower trypsin inhibitor-1 (SFTI-1) and the cyclotide kalata B1 (Table 1). 

SFTI-1 is one of the smallest cCPPs and a head-to-tail cyclic peptide, with a single disulphide bond [25]. From a drug-design perspective, SFTI-1 has several highly desirable properties that are atypical for small peptides [26]. These include proteolytic stability, relatively small molecular weight, and possibility of use as a scaffold combined with reported high cell-penetration ability with a yet undefined entry mechanism [26]. Of note, several α_v_β_6_ integrin binding SFTI-1-based peptides have been synthesized as potential theranostic agents for head and neck squamous cell carcinoma [27]. Roesch et al. showed that one of the SFTI-1-based peptides, SFLAP3, demonstrated high stability and target specificity as well as the ability to accumulate in primary and metastatic tumours, also confirmed in patients using positron emission tomography [27]. Hence, the improvement of BBB specificity of grafted SFTI-1 analogues may unravel novel brain drug-delivery opportunities. 

Kalata B1 is a cell-penetrating cyclotide from *Oldenlandia affinis*, with head-to-tail cyclization and a cyclic cystine knot structure stabilized by three di-sulphide bonds [28]. The peptide is reported to enter the cell via membrane interactions leading to vesicular uptake [29]. Remarkably, it is internalized via non-toxic and non-permeabilizing mechanisms as efficiently as the gold-standard transactivating transcriptional activator peptide [30]. If the uptake mechanism is also preserved in brain endothelial cells, it could be utilized for the delivery of cargo across the BBB. 

The permeability features of SFTI-1 and kalata B1 have previously been studied using synthetic membranes and in models of peripheral cell types [29,30,31,32,33]. However, their cell-penetrating properties have not yet been investigated in detail in the brain context. The lack of knowledge hampers their potential application for CNS drug delivery. In fact, the information on in vivo neuropharmacokinetics (neuroPK) of cCPPs in general, and even on systemic pharmacokinetics (PK) of cCPPs, is limited. Their cell-penetration capability should be confirmed by in vivo studies to guide future drug design. In this regard, one of the important concerns is related to the methodologies used to characterize neuroPK of cCPPs, as the conclusions made hinge on the sensitivity and specificity of the method used.

In the present study, we aimed to define and characterize the PK and neuroPK of SFTI-1 and kalata B1, to test their suitability as scaffolds for drug delivery into the brain, and, more broadly, to characterize their behaviour in vivo. We used complementary approaches of an in vivo rat model, a human in vitro BBB model, a mathematical simulation, and various tissue assays to describe the extent and rate of BBB transport of these peptides and their within-brain partitioning. The analysis revealed that SFTI-1, but not kalata B1, is a potential CNS delivery scaffold for drugs directed to brain extracellular targets. 

## 2. Materials and Methods

Data and associated protocols will be available to readers without undue qualifications in material transfer agreements. Bioanalysis of samples using a liquid chromatography–tandem mass spectrometry (LC-MS/MS) method was performed according to general guidelines from FDA requirements on the validation of bioanalytical methods, when applicable [34]. Reporting of animal studies was performed in agreement with ARRIVE (Animal Research: Reporting of In Vivo Experiments) guidelines [35].

### 2.1. Materials

Acetonitrile, methanol, and formic acid were of analytical grade (Merck, Darmstadt, Germany). Trifluoroacetic acid (TFA), disodium phosphate, sodium dihydrogen phosphate, sodium chloride, potassium chloride, magnesium sulphate heptahydrate, potassium phosphate dibasic trihydrate, calcium chloride dihydrate, ascorbic acid, sodium hydroxide, and glucose were obtained from Merck (Darmstadt, Germany). HEPES (4-(2-hydroxyethyl)-1-piperazineethanesulfonic acid), monensin sodium, dynasore hydrate, dimethyldichlorosilan in heptane, dichloromethane, foetal bovine serum, and agarose Type VII-A were purchased from Sigma-Aldrich (Steinheim, Germany). Sterile saline was purchased from Fresenius Kabi (Bad Homburg, Germany). Endothelial cell Growth Medium-2 (EGM-2) was obtained from Lonza (Walkersville, MD, USA). Vascular endothelial growth factor 165, basic fibroblast growth factor, was obtained from PeproTech (Rocky Hill, CT, USA). Dulbecco’s Modified Eagle’s Medium was obtained from Life Technologies (Paisley, UK). Gentamycin was obtained from Biochrom AG (Berlin, Germany). Endothelial cell medium (ECM-5) and endothelial cell growth factor were obtained from Sciencell (Carlsbad, CA, USA). RLT Lysis buffer was obtained from Qiagen (Germantown, MD, USA). Water was purified with a Milli-Q Academic system (Millipore, Bedford, MA, USA). All plastic materials were tested for sticking, and low-binding microtubes were used for all studies (Sarstedt, Nümbrecht, Germany).

### 2.2. Peptide Production

SFTI-1 was extracted according to the following protocol: 400 g of seeds from *Helianthus annuus*, common sunflower, was crushed and extracted with 1 L 60% acetonitrile for 3 h. The mixture was filtered, and the seeds were re-extracted in fresh solvent for an additional 3 h. Following filtration, the extracts were pooled and the majority of acetonitrile was removed by rotary evaporation. The extract was separated from lipids through partitioning against dichloromethane (1:1, *v*:*v*). The aqueous layer was freeze-dried, dissolved in MilliQ-water with 10% acetonitrile and 0.05% TFA, and subjected to solid-phase extraction. A C18 Isolute cartridge (Biotage, Uppsala, Sweden) was conditioned in methanol and equilibrated with 10% acetonitrile and 0.05% TFA in MilliQ-water. The extract was loaded, washed with 0.05% TFA in MilliQ-water, and eluted with increasing concentrations of acetonitrile in MilliQ-water with 0.05% TFA. The SFTI-1-containing fractions were pooled, diluted to 5% acetonitrile in 0.05% TFA, and was purified using reverse-phase HPLC (C18, 250 × 10 mm, 5 µm, 300 Å, Phenomenex, Torrance, CA, USA). The purity and identity of SFTI-1 were analysed using HPLC-UV (Shimadzu, Tokyo, Japan) and UPLC-MS/MS (Waters, Milford, MA, USA), Figure S3. Kalata B1 was extracted and purified from the leaves of *Oldenlandia affinis* with the same procedure as SFTI-1 using a similar protocol to one described previously [36]. Both peptides were >95% pure.

### 2.3. Animals

Male Sprague Dawley rats weighing 250–300 g (Taconic, Lille Skensved, Denmark, n = 53) were used in all experiments. The animals were housed in groups with ad libitum access to food and water and with a 12 h light–dark cycle and were allowed to acclimatize for seven days before the start of this study. All animal experiments were performed in accordance with the guidelines from the Swedish National Board for Laboratory Animals and were approved by the Animal Ethics Committee of Uppsala, Sweden (Ethical approval C188/14 and C16/12). 

### 2.4. Assessment of Systemic Pharmacokinetics in Rats

**Figure 1:**
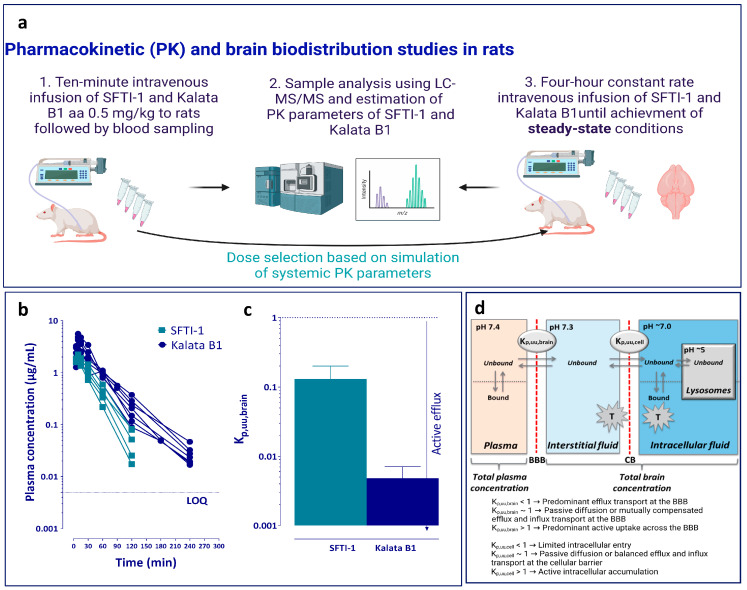
Pharmacokinetic studies with a focus on the fate of cCPPs in blood and the extent of BBB transport in rats. (**a**) Overview of pharmacokinetic studies focused on the assessment of systemic parameters after a 10 min intravenous infusion of peptides and brain distribution after a 4 h constant-rate intravenous infusion with the achievement of steady-state conditions. (**b**) Total plasma concentration–time profiles of SFTI-1 (turquoise, n = 5) and kalata B1 (blue, n = 7) after a 10 min infusion of Sprague Dawley male rats with 0.5 mg/kg of the respective peptide. Data for individual rats are shown. All data points for SFTI-1 post 120 min time point were below the quantification limit, i.e., 5 ng/mL. (**c**) The extent of blood–brain barrier (BBB) transport of SFTI-1 (turquoise) and kalata B1 (blue), assessed using the unbound brain-to-plasma concentration ratio, K_p,uu,brain_. Data are presented as the mean + SD (n = 6 rats for each cyclotide) and assessed using the propagation of uncertainty method (see, Section 2.12. (**d**) Key compartments involved in brain disposition of peptides with the essential pharmacokinetic parameters describing the extent of BBB transport, K_p,uu,brain_, and the extent of cellular barrier (CB) transport, K_p,uu,cell_, indicated.

An overview of the pharmacokinetic studies is presented in Figure 1a. Since previous pharmacokinetic data were extremely sparse, a pilot study was performed in rats (n = 2), and simulations were performed in Berkeley Madonna (version 8.3.18 for Windows, Berkeley, CA, USA), defining suitable dose and sampling intervals from the parameters obtained in the pilot study. The surgical implantation of catheters made of polyethylene (PE50) in the femoral vein and artery for dosing of peptide and sampling of blood, respectively, was performed the day before experiments under isoflurane anaesthesia. The animals were then housed in a CMA120 system (CMA, Solna, Sweden) for freely moving animals with ad libitum access to food and water. To determine the systemic pharmacokinetic parameters of the two peptides, a 10 min infusion was administered at a dose of 0.5 mg/kg in saline (n = 5 for SFTI-1 and n = 7 for kalata B1). Blood samples (200 µL) were taken from the femoral artery at 0, 5, 9, 15, 30, 60, 90, 120, and 180 min for SFTI-1 and 0, 5, 9, 15, 30, 60, 90, 120, 180, and 240 min for kalata B1. All samples were added to heparinized low-binding Eppendorf tubes. The blood samples were centrifuged, and plasma was transferred to new Eppendorf tubes and immediately frozen at −20 °C. After bioanalysis, the area under the plasma concentration–time curve (AUC) was determined by the trapezoidal rule with extrapolation to time infinity followed by calculation of systemic clearance (CL), apparent volume of distribution (V_d_), and half-life (t½).

### 2.5. Assessment of Key Neuropharmacokinetic Parameters Including BBB Transport in Rats

In order to assure the accurate assessment of the extent of BBB transport and to minimize the use of animals, the achievement of steady-state conditions of total plasma concentration and equilibration across the blood–brain interface are critical [37]. This was achieved by combining a short 5 min fast-rate infusion (60 µg/min/kg) to reach targeted total plasma concentrations quicker, followed by a constant slow-rate infusion (2.12 µg/min/kg) over 4 h, at a total dose of 0.5 mg/kg (n = 6 per peptide). The infusion was administered through the catheter placed in the femoral vein. Blood (200 µL) was sampled from the catheter in the femoral artery at 1, 2, and 3 h into heparinized tubes to ensure that steady-state conditions were obtained in plasma. At 4 h, blood was sampled through heart puncture using EDTA vacutainers (BD, Eysins, Switzerland), and the whole brain was collected. The blood samples were centrifuged, and plasma was collected. All samples were immediately frozen on dry ice and then kept at −20 °C pending analysis.

The in vivo total brain-to-plasma concentration ratio, K_p,brain_, was studied by measuring steady-state concentrations in plasma and the total amount in the brain (Equation (1)).

(1) $$K_{p,\mathrm{brain}}=\frac{A_{\mathrm{brain}}}{C_{\mathrm{plasma}}}$$

where A_brain_ is the total amount of peptide in the brain calculated using Equation (2), including compensation for the residual cerebral blood [38], and C_plasma_ is total plasma concentration (µg/mL).

(2) $$A_{\mathrm{brain}}=\frac{C_{\mathrm{brain}}-V_{\mathrm{eff}}\times C_{\mathrm{plasma}}}{1-V_{\mathrm{water}}}$$

where C_brain_ (µg/g brain tissue) is the concentration of drug in the brain homogenate, V_eff_ is the effective plasma space, 10.1 µL/g brain, and V_water_ is the volume of plasma water, 10.3 µL/g brain [38]. Potential accumulation of peptides in the endothelial cells was tested using the capillary depletion method, which showed no detectable levels of peptides in brain microvasculature fraction. The blood-to-plasma ratio was assessed, and no accumulation in blood cells was observed.

### 2.6. Assessment of Uptake into Brain and Lung Slices

The brain slice assay was performed according to previously published protocols [39,40]. Three animals per study group were investigated. All glassware used in the brain slice experiments was silanized using dimethyldichlorosilane in heptane to avoid non-specific binding of the cyclic peptides to glass. In short, the silanization solution was added to the glassware and left there for one hour, after which the glassware was rinsed with methanol and then placed in a heating cabinet to cure. Slicing was performed with a Leica VT1200 microtome slicer (Leica Microsystems, Wetzlar, Germany). In short, to measure the binding and uptake, six 300 µm thick brain slices from the rostral striatal area of each brain were incubated in 15 mL of artificial extracellular fluid (aECF, pH 7.4 at 37 °C) spiked with individual peptide at a concentration of 200 nM (302 ng/mL for SFTI-1 and 578 ng/mL for kalata B1). Beakers were placed in a MaxQ4450 shaker (Thermo Fisher Scientific, NinoLab, Sweden) for 5 h at 45 rpm and 37 °C with constant oxygenation. At the end of the incubation, two 200 µL samples of aECF were taken and mixed with blank brain homogenate (1:4, *w*:*v*) for assessment of unbound buffer concentration, C_buffer_ (nanomoles per mL). Thereafter, the brain slices were individually sampled, carefully dried on filter paper, weighed, and homogenized in aECF (1:9, *w*:*v*) to determine the total amount of drug in the slice, A_slice_ (nanomoles per g brain). The density of brain tissue was assumed to be 1 g/mL. The key assumption of the assay is achievement of equilibrium during 5 h incubation, with C_buffer_ being equal and representing unbound brain slice interstitial concentration. Samples of aECF (200 µL) were also taken from the scintillation vial without brain slices at the start and at the end of the incubation to determine the relative recovery and thermostability of the peptides. The unbound volume of distribution in brain, V_u,brain_ (mL/g brain), was calculated using Equation (3).

(3) $$V_{u,\mathrm{brain}\;}=\frac{A_{\mathrm{slice}}-V_{i}\times C_{\mathrm{buffer}}}{C_{\mathrm{buffer}}\left(1-V_{i}\right)}$$

where V_i_ is the volume of buffer film that coats the slice and equals to 0.094 mL/g brain [39].

**Figure 2:**
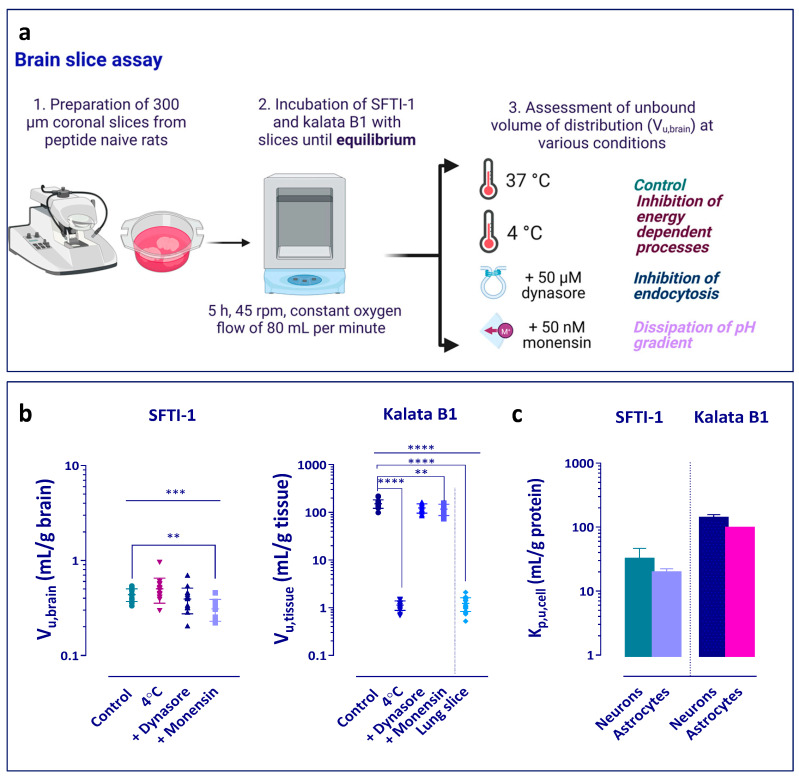
Tissue distribution characteristics of SFTI-1 and kalata B1. (**a**) Overview of the brain slice assay. (**b**) Unbound volume of distribution of SFTI-1 and kalata B1 in the brain, V_u,brain_ (mL/g brain). Several potential peptide uptake mechanisms were explored in the brain slice assay under control conditions at 37 °C, and by (i) incubation of peptides at 4 °C to investigate the contribution of energy-dependent routes; (ii) pre-incubation with the dynamin inhibitor dynasore (50 µM) to examine the involvement of endocytic pathways; and (iii) pre-incubation with the ionophore monensin (50 nM) to evaluate the impact of pH-dependent processes. Properties of kalata B1 were also tested using lung slices. Data were analysed using one-way ANOVA and Dunnett’s multiple comparisons test. Note the differences in scale between SFTI-1 and kalata B1 data. (**c**) In vitro cellular distribution of SFTI-1 and kalata B1, presented as the total cell-to-unbound medium ratio, K_p,u,cell_ (mL/g total protein), assessed after 5 h incubation with neurons and astrocytes differentiated from human neural stem cells. Data are presented as the mean ± SD, with *p* < 0.0001 (****), *p* < 0.001 (***), *p* < 0.01 (**). Each experiment includes 3–6 independent experiments.

**Figure 3:**
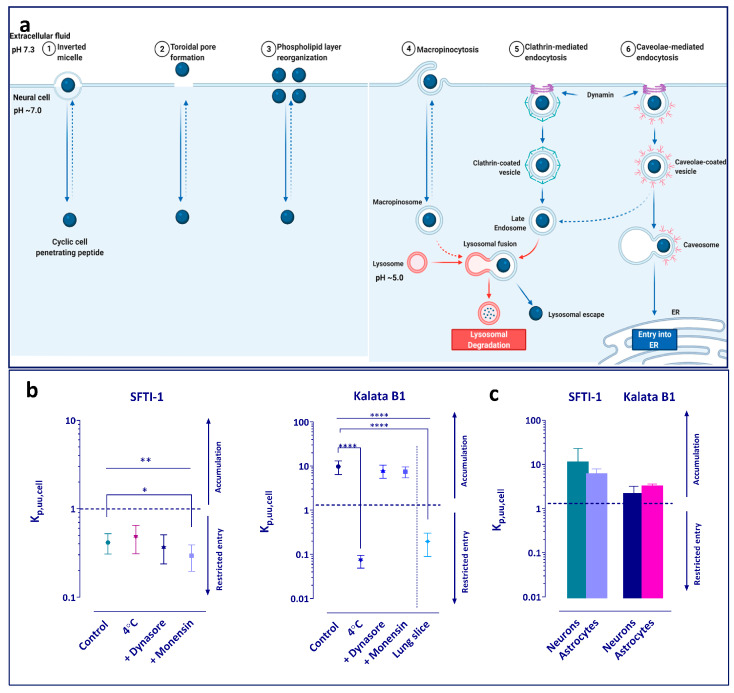
Intracellular uptake characteristics of SFTI-1 and kalata B1. (**a**) Plausible peptide cell entry mechanisms, including direct translocation (1–3) and endocytic pathways (4–6), reviewed in [14]. (**b**) Unbound intracellular-to-extracellular concentration ratio, K_p,uu,cell_, of SFTI-1 and kalata B1, determined in brain and lung slices. Data were analysed using one-way ANOVA and Dunnett’s multiple comparisons test. Note the differences in scale between SFTI-1 and kalata B1 data. (**c**) Unbound intracellular-to-extracellular concentration ratio, K_p,uu,cell_, of SFTI-1 and kalata B1, determined in neurons and astrocytes that had been differentiated from human neural stem cells. Data are presented as the mean ± SD, assessed using the propagation of uncertainty method (see Section 2.12) with *p* < 0.0001 (****), *p* < 0.01 (**), *p* < 0.05 (*).

To study the potential mechanism of intracellular distribution of the peptides, several exploratory experiments were performed (Figure 2a). Initially, the brain slice assay was performed at 4 °C to elucidate whether any active processes were involved in the uptake of the peptides. In addition, the role of endocytosis and pH partitioning-driven distribution was investigated. The slices were pre-incubated for 30 min with the blockers before the addition of the peptides. Dynasore was used as an inhibitor of dynamin-dependent endocytosis, which is involved in both clathrin and caveolin vesicle formation [41] (Figure 3a). A concentration of 50 µM was chosen as it has previously been shown to provide a 70% decrease in dynamin activity and is more than three-fold higher than the IC50 value of 15 µM [41,42]. Monensin was used as an ionophore modifying the pH of the various cellular compartments towards increasing pH, hence decreasing the trapping of compounds in acidic cellular sub-compartments [43,44,45]. Due to high cytotoxicity, the concentration of 50 nM was chosen based on previous studies [43].

As a comparative tissue, and in order to examine whether the uptake of kalata B1 is a brain-specific phenomenon, lung slice experiments were performed according to Bäckström et al. [46]. In short, lung slices were prepared by performing an in situ perfusion of the lungs of the rats with physiological saline and then filling them with agarose Type VII-A to facilitate slicing. Slices of 500 µm thickness were prepared using the Leica VT1200 microtome slicer (Leica Microsystems, Wetzlar, Germany). Three slices (n = 3 biological replicates) were incubated in 15 mL of buffer containing kalata B1 with an initial concentration of 200 nM. After a 5 h incubation at 37 °C in an incubated shaker (MaxQ4450 Thermo Fisher Scientific, NinoLab, Stockholm, Sweden) with a rotation speed of 45 rpm, the buffer and lung slices were sampled. To match the matrix of the lung slice samples, 200 μL of aECF was mixed with 200 μL of blank agarose-filled lung homogenate (1:4, *w*:*v*). The lung slices were individually removed, dried on filter paper, weighed, and thereafter homogenized in 9 volumes (*w*:*v*) of aECF with an ultrasonic processor (VCX-130; Sonics, Chemical Instruments AB, Stockholm, Sweden). All samples were stored at −20 °C pending LC-MS/MS analysis. Assuming that at equilibrium the concentration of the peptide in virtually protein-free buffer is equal to the interstitial fluid concentration in the lung slice, the V_u,lung_ (mL/g lung) was estimated using Equation (4) as a ratio of the amount of compound in the lung slice (A_lung_, nanomoles g lung) to the measured final buffer concentration (C_buffer_, micromole/L).

(4) $$V_{u,\mathrm{lung}}=\frac{A_{\mathrm{lung}}-V_{i}\times C_{\mathrm{buffer}}}{C_{\mathrm{buffer}}\times \left(1-V_{i}\right)}$$

where V_i_ (mL/g lung) is the volume of the surrounding lung slices and air spaces layer of buffer. A V_i_ volume of 0.73 mL/g lung was used [46].

### 2.7. Evaluation of Tissue and Cell Binding Properties In Vitro

Peptide binding to plasma proteins, brain tissue homogenate, and lung tissue homogenate as well as to neuronal and astrocyte cell culture homogenates was investigated using equilibrium dialysis [47]. Undiluted plasma and brain from three peptide-naïve rats were used in the experiment. The brain homogenate was prepared by homogenizing 1 part brain with 4 parts of phosphate-buffered saline (PBS, pH 7.4). Lung homogenate was prepared by homogenizing agarose-filled lungs (obtained from lung slice experiments) with four volumes (*w*:*v*) of PBS buffer. Neurons and astrocytes differentiated from human neural stem cells (hNSCs) seeded at a density of 2.5 × 10^4^ cells/cm^2^ in 6-well plates were lysed with 300 µL of Pierce Lysis buffer and used for determination of the binding of SFTI-1 and kalata B1 to the cultured cells. Semi-permeable membranes with a molecular weight cut-off of 12–14 kDa were used and placed in an HTD96b apparatus (HTDialysis, Gales Ferry, CT, USA). The undiluted plasma and respective homogenates were spiked with individual peptide to a concentration of 1 µM and were dialyzed against PBS (pH 7.4). Five technical replicates were prepared per biological replicate. The experiment was run for 6 h at 37 °C in a MaxQ4450 shaker (Thermo Fisher Scientific, NinoLab, Sweden) at 200 rpm. The time to reach equilibrium has been tested in pilot experiments, and 6 h was found to be sufficient for achievement of equilibrium. Aliquots (80 µL) were taken from the buffer and plasma/homogenate sides. To compensate for any differences in matrix during bioanalysis, the plasma and homogenate samples were mixed with equal volumes of blank buffer, and the buffer samples were mixed with an equal volume of relevant blank plasma/homogenate. Spiked plasma and respective homogenate samples were incubated to control for relative recovery and thermal stability of the peptides in respective matrices.

The unbound fraction in undiluted plasma was calculated using Equation (5), where C_buffer_ is the unbound concentration of peptide in the buffer and C_plasma_ is the total concentration of peptide in plasma at 6 h. The binding of peptide in various homogenates was assessed in two steps. First, the fraction of unbound peptide in the diluted homogenate, f_u,h,D_, was calculated using Equation (6), where C_homogenate_ is the total concentration of peptide in respective homogenate at 6 h. Second, the correction for the dilution was implemented as described in Equation (7), where D is the dilution factor used in the preparation [47]. D was 5 for brain and lung homogenates and on average 35.3 for the cell culture studies, as determined based on the total protein content and the total volume of lysate.

(5) $$f_{u,\mathrm{plasma}}=\frac{C_{\mathrm{buffer}}}{C_{\mathrm{plasma}}}$$

(6) $$f_{u,h,D}=\frac{C_{\mathrm{buffer}}}{C_{\mathrm{homogenate}}}$$

(7) $$f_{u,\mathrm{tissue}/\mathrm{cell}}=\frac{\frac{1}{D}}{\left(\left(\frac{1}{f_{u,h,D}}\;\right)-1\right)+\frac{1}{D}}$$

### 2.8. Evaluation of BBB Permeably in Human In Vitro BBB Model

The in vitro BBB model, consisting of CD34^+^ endothelial cells (ECs) derived from hematopoietic stem cells in co-culture with bovine brain pericytes, was established as described by Cecchelli et al. [48]. Written and informed consent was obtained for the collection of human umbilical cord blood, in compliance with French legislation. The protocol was approved by the French Ministry of Higher Education and Research (reference; CODECOH DC2011-1321). All experiments were carried out in line with the authorized protocol.

**Figure 4:**
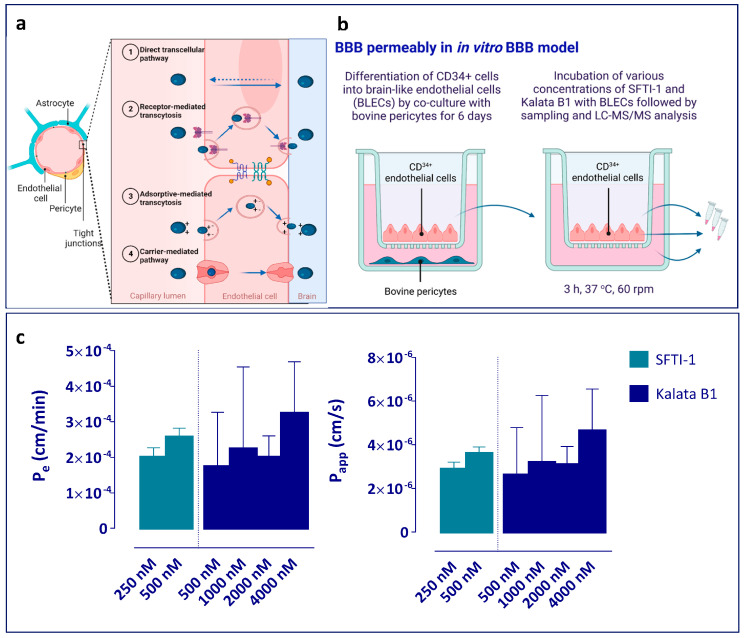
Permeability studies characterizing the rate of BBB transport in a human CD34^+^ in vitro BBB model. (**a**) Key blood–brain barrier (BBB)-forming cells and potential mechanisms of peptide transport across endothelial cells. (**b**) Overview of the setup for the investigation of peptide permeability of brain-like endothelial cells in vitro. Sodium fluorescein was used as a marker of cell monolayer integrity. cCPPs in all samples were analysed using LC-MS/MS. (**c**) Endothelial permeability (P_e_, cm/min) and apparent permeability (P_app_, cm/s) of SFTI-1 (turquoise, n = 3 independent experiments) and kalata B1 (blue, n = 3 independent experiments). No significant differences between the tested concentrations of individual peptides nor between two peptides were detected by ANOVA analysis. Data are presented as the mean + SD.

These isolated CD34+ cells were cultured in EGM-2, supplemented with 20% (*v*:*v*) FBS and 50 ng/mL vascular endothelial growth factor on 1% (*w*:*v*) gelatine-coated 24-well plates (2 × 10^5^ cells/well). After 15–20 days, ECs are seen in the culture dish. For each experiment, the cells were expanded in 1% (*w*:*v*) gelatine-coated T75 flasks (BD Biosciences, Le Pont-de-Claix, France) in EGM-2. Bovine brain pericytes were isolated and characterized as described by Vandenhaute et al. [49]. Sub-clones were cultured in Dulbecco’s Modified Eagle’s Medium supplemented with 20% (*v*:*v*) FBS, 2 mM L-glutamine, 50 µg/mL gentamycin, and 1 ng/mL basic fibroblast growth factor. At confluency, 5 × 10^4^ cells were seeded in ECM-5 with 5% (*v*:*v*) FBS, 50 µg/mL gentamycin, and 0.5% endothelial cell growth factor in gelatine-coated 12-well plates (Costar, Corning Inc., New York, NY, USA). CD34^+^-derived ECs were seeded at 8 × 10^4^ cells per Matrigel-coated (BD Biosciences) insert (3401, Corning Inc., NY, USA) and put in co-culture with the bovine brain pericytes, with the pericytes at the bottom of the well, for 6 days to achieve brain-like endothelial cell (BLEC) characteristics (Figure 4b).

For the BLEC permeability experiments, kalata B1 and SFTI-1 were dissolved in Ringer–HEPES (RH) buffer (NaCl 150 mM, KCl 5.2 mM, CaCl_2_ 2.2 mM, MgCl_2_ 0.2 mM, NaHCO_3_ 6 mM, Glucose 2.8 mM, HEPES 5 mM, water for injection, pH 7.4), supplemented with 0.5% (*w*:*v*) human serum albumin to final concentrations of 250 nM and 500 nM for SFTI-1 and 250 nM, 500 nM, 1000 nM, 2000 nM, and 4000 nM for kalata B1.

At the beginning of the permeability experiments, inserts with BLEC monolayers were transferred to new abluminal (receiver) compartments previously filled with 1.5 mL of RH buffer, and 500 µL of the compounds dissolved in RH buffer, with the addition of the paracellular marker sodium fluorescein (NaFlu), were added to the luminal (donor) compartment. After 3 h of incubation at 37 °C, with a shaking velocity of 60 rpm, aliquots were taken from the donor and receiver compartment. The BLEC monolayers were washed twice with cold RH buffer and lysed with RLT Lysis buffer. The samples were kept at −20 °C pending analysis in low-binding tubes.

To evaluate the tightness of the BLEC monolayer and the effect of the peptides (kalata B1 or SFTI-1) on the integrity of the BBB, the permeability to NaFlu was assessed. An increase in the permeability to NaFlu in the presence of the peptide can indicate an increase in paracellular leakage and consequent loss of barrier integrity. To evaluate the ability of test compounds to cross Matrigel-coated filters without cells and to control for the adsorption to plastics and/or Matrigel, the NaFlu and peptide’s permeability over inserts without cells were also assessed. Mass balance (M.B%) was calculated from the amount of compound recovered in both compartments at the end of the experiment divided by the total amount added to the donor compartment at the start of the experiment.

Samples from both compartments as well as the lysed endothelial cells were analysed for fluorescence using a Synergy H1 (Biotek) set to quantify NaFlu (wavelengths Excitation 490 nm and Emission 525 nm) and with UPLC-MS/MS to assess the permeability and cellular uptake of the peptides. All experiments were performed in triplicate (n = 3 independent experiments per concentration and cCPPs).

The raw data were computed to generate the endothelial permeability (P*_e_*, cm/min) and the apparent permeability (P_app_, cm/s) according to the following equations:

(8) $$\frac{I}{P_{\mathrm{se}}}=\frac{1}{P_{\mathrm{st}}}-\frac{1}{P_{\mathrm{sf}}}$$

(9) $$P_{e}=\frac{P_{\mathrm{se}}}{S}$$

(10) $$P_{\mathrm{app}}=\frac{J}{S\times C_{0}}$$

where P_se_ is the permeability surface area product across the endothelial cell layer, P_st_ is the total permeability surface area product across the endothelial cell layer and coated-filter insert, P_sf_ is the permeability surface area product across the coated-filter insert, and S is the surface area of the Transwell insert (i.e., 1.12 cm^2^). P_app_ is the apparent permeability, J is the rate of appearance of the compound in the receiver compartment (amount/sec), and C_0_ is the concentration in the donor compartment at the start of the experiment (amount/mL). The high recovery of Naflu and peptides at the end of the transport experiments without cells (M.B% superior to 95%) indicated that the non-specific binding of the compounds to cell culture material in these experiments was negligible.

### 2.9. Evaluation of Uptake of Peptides in Cell Cultures

Human neural stem cells (hNSCs) derived from human embryonic stem cells (hESCs) were differentiated to neurons or astrocytes [50]. The cell lines were seeded at a density of 2.5 × 10^4^ cells/cm^2^ in triplicates on a 6-well plate, with one well of control, where no peptide was added to the medium. Cells were allowed to differentiate into desired lineage for three weeks before the addition of peptides. The peptides were individually added to 2 mL Neurobasal medium to a final concentration of 500 nM and incubated at 37 °C for 5 h. The medium was removed, and the cells were subsequently lysed using 300 µL of Pierce™ lysis buffer (ThermoFisher Scientific Cat. No. 87787) per well. Samples were kept at −20 °C pending analysis. The uptake of peptides was determined using the total cellular-to-unbound medium concentrations ratio, K_p,u,cell_ (mL/g total protein), according to Equation (11).

(11) $$K_{p,u,\mathrm{cell}}=\frac{C_{\mathrm{cell}}}{C_{u,\mathrm{medium}}}=\frac{A_{\mathrm{cell}}/V_{\mathrm{cell}}}{C_{u,\mathrm{medium}}}$$

where A_cell_ (nanomoles per g total protein) is the total amount of peptide in the cell lysate normalized by the total protein content and estimated volume of the cells. Total protein concentration (mg/mL) in the cell lysate was determined using a NanoDrop 2000C (ThermoFisher, Waltham, MA, USA) by measuring the absorbance at 280 nm. V_cell_ was estimated using the value of 6.5 µL/mg protein by analogy of HEK293 cells [51]. C_u,medium_ (nanomoles per mL) is the unbound concentration of peptide in the medium at the end of 5 h incubation, quantified by UPLC-MS/MS (NB: nonspecific binding was neglected). The parameter K_p,u,cell_ (mL per g of total protein at 5 h) is a partition coefficient between the buffer and the cell culture, where the fraction in the buffer has no binding, but the measured concentration in the cells is a composite of both uptake into the cells and binding to both the cell surface and intra-cellular components. This parameter is thereby similar to V_u,brain_ as it also gives a description of both binding and uptake processes.

For estimation of the extent of transport across the cellular membrane, the unbound intracellular-to-extracellular ratio, K_p,uu,cell_ was calculated as described below:

(12) $$K_{p,\mathrm{uu},\mathrm{cell}}=K_{p,u,\mathrm{cell}}\times f_{u,\mathrm{cell}}$$

### 2.10. Bioanalysis

All samples, standards, quality controls, and blanks in respective matrices were prepared by protein precipitation. Fifty µL of sample was precipitated with 150 µL of acetonitrile. The samples were vortexed and centrifuged for three minutes at 10,000 rpm in a Scanspeed mini (Labogene, Lynge, Denmark). The supernatants, 150 µL, were then transferred to new low-binding tubes and evaporated to dryness under N_2_ at 40 °C. The dried samples were then re-dissolved in 100 µL 20% acetonitrile with 0.05% formic acid in MilliQ water. Samples were then transferred to glass LC autosampler vials and kept at 5 °C until analysis.

Kalata B1 samples were analysed according to the method described in Melander et al., 2016 [52]. The analytical method for SFTI-1 was developed in the same manner. The separation of analyte was performed on either LC-10ADvp pumps and a SIL-HTc autosampler (Shimadzu, Kyoto, Japan) connected to a HyPurity C18 column (50 × 4.6 mm, particle size 3 µm), protected by a guard-column of the same material (10 × 4.0 mm, particle size 3 µm) (Thermo Scientific, Waltham, MA, USA), or an Acquity UPLC (Waters, Milford, MA, USA) connected to a Peptide CSH C18 column (50 × 2.1 mm, particle size 1.7 µm) (Waters, MA, USA). Two mobile phases, A and B, with the following compositions were used for the gradient in the separation, mobile phase A: 90% MilliQ water, 10% acetonitrile, and 0.05% formic acid; mobile phase B: 10% MilliQ water, 90% acetonitrile, and 0.05% formic acid. A full description of the gradients can be found in the Supplementary Materials (Table S1).

A Quattro Ultima Micromass triple quadrupole mass spectrometer (Waters, Milford, MA) as well as a Xevo TQ-S Micro triple quadrupole mass spectrometer (Waters, Milford, MA) were used for the quantification of the peptides. The positive ionization mode was used in the electrospray ionization, and the analysis was performed using multiple reaction monitoring (MRM). The monitored mass pairs were 1446.8/1446.8 *m/z* for kalata B1 and 757.56/757.56 *m/z* for SFTI-1. The desolvation temperature was set to 500 °C, and the source temperature was 150 °C. A full set of analytical parameters can be found in the Supplementary Materials (Table S2). All data were analysed using MassLynx v.4.1 or v.4.2 (Waters, Milford, MA, USA). The linear range of the analytical method was 5–10,000 ng/mL in plasma and 5–5000 ng/g in brain homogenate for SFTI-1. For kalata B1, it was 2–10,000 ng/mL in plasma and 5–5000 ng/g in brain homogenate. In lung homogenate, the linear range was 5–1000 ng/g, and in cell lysates, the linear range of the method was 5–2000 ng/mL for both peptides. The lowest point in the respective standard curve was set as the limit of quantification. QC-levels were set to 12, 120, and 1200 ng/mL in plasma and 7.5, 75, and 750 ng/g in brain, where the QCs were within ±15% of the nominal value. Standard curves had to have an R^2^-value of >0.99 to be accepted, a weighting factor of 1/x^2^ was used, and the curve was not forced through the origin.

### 2.11. Estimation of the Extent of BBB and Cellular Barrier Transport

The unbound brain-to-plasma ratio, K_p,uu,brain_, was calculated using Equation (13) according to the CMA [37], where K_p,brain_ is the total brain-to-plasma ratio, f_u,plasma_ is the unbound fraction in plasma as measured with equilibrium dialysis, and V_u,brain_ is the unbound volume of distribution in brain measured with the brain slice technique.

(13) $$K_{p,\mathrm{uu},\mathrm{brain}}=\frac{K_{p,\mathrm{brain}}}{f_{u,\mathrm{plasma}}\times V_{u,\mathrm{brain}}}$$

The unbound intracellular-to-extracellular concentration ratio, K_p,uu,cell_, was calculated according to Equation (14) by combining the brain slice derived, V_u,brain_, with the measurement of brain tissue binding, f_u,brain_, from the tissue homogenate study [53]. This allows elucidation of whether the uptake into the brain cells is driven mainly by passive processes and intracellular binding and/or partitioning, such as lysosomal trapping, or by active uptake into the cells.

(14) $$K_{p,\mathrm{uu},\mathrm{cell}}=f_{u,\mathrm{brain}}\times V_{u,\mathrm{brain}}$$

In addition, a simulation exercise was performed to evaluate the unbound brain exposure of SFTI-1 and kalata B1 using Berkeley Madonna (version 8.3.18 for Windows, Berkeley, CA, USA). The simulation was performed at given initial total plasma concentration to be equal to steady-state concentrations obtained in the neuroPK study (Table 2), utilizing the parameters listed in Supplementary Material Table S3. A two-compartment model consisting of blood and brain compartments with a focus on unbound concentrations was characterized (Supplementary Material Figure S1 describes the structure of the model and the codes).

### 2.12. Statistical Analysis

Statistical analysis was performed using GraphPad Prism v6.07 (GraphPad Software, San Diego, CA, USA). Permeability data were analysed with one-way ANOVA with Tukey’s multiple comparisons test, normality was tested using the D’Agostino–Pearson test. Differences were considered to be significant when *p*-values were <0.05. Brain slice and K_p,uu,cell_ data were analysed with one-way ANOVA with Dunnett’s multiple comparisons test. Differences in cellular uptake were analysed with an unpaired *t*-test. Illustrations are prepared using BioRender (Toronto, ON, Canada).

Composite parameters (K_p,uu,brain_, K_p,uu,cell_) were calculated using the propagation of error method in order to obtain standard deviations [54,55]. As the K_p,uu,brain_ and K_p,uu,cell_ pharmacokinetic parameters were not a result of a direct single measurement but obtained in two or three steps, the assessment of uncertainty also involved those steps [54]. Propagation of uncertainty was assessed for both product and quotient of two variables, A and B, using the following equations.

Propagation of uncertainty of K_p,uu,cell_ was performed using the product rule. Let A and B be variables with standard deviations $\sigma_{A}$ and $\sigma_{B}$ and set

(15) f=A×B.

Propagated uncertainty for a product, i.e., the standard deviation of f, was then calculated as follows:

(16) $$\sigma_{f}\approx \left|f\right|\sqrt{{\left(\frac{\sigma_{A}}{A}\right)}^{2}+{\left(\frac{\sigma_{B}\;}{B}\right)}^{2}+2\frac{\sigma_{\mathrm{AB}}}{\mathrm{AB}}}$$

The covariance $\sigma_{\mathrm{AB}}$ was then calculated with help of the correlation r as $\sigma_{\mathrm{AB}}=r\cdot \sigma_{A}\cdot \sigma_{B}$.

Propagation of uncertainty of K_p,uu,brain_ was performed using the quotient rule. Let A and B be variables with standard deviations $\sigma_{A}$ and $\sigma_{B}$ and set

(17) $$f=\frac{A}{B}$$

Propagated uncertainty for a quotient, i.e., the standard deviation of f, was then calculated as follows:

(18) $$\sigma_{f}\approx \left|f\right|\sqrt{{\left(\frac{\sigma_{A}}{A}\right)}^{2}+{\left(\frac{\sigma_{B}}{B}\right)}^{2}-2\frac{\sigma_{\mathrm{AB}}}{\mathrm{AB}}}$$

As above, the covariance was calculated as $\sigma_{\mathrm{AB}}=r\cdot \sigma_{A}\cdot \sigma_{B}$.

## 3. Results

### 3.1. Low Extent of BBB Transport of SFTI-1 and Kalata B1 In Vivo

In accordance with the foundational free-drug theory, it is only the unbound (free) drug that crosses the cellular barrier, including the BBB, and elicits an effect [56,57,58]. Consequently, the unbound drug concentrations in the blood and the brain are the most relevant measures when studying the neuroPK and pharmacodynamics of a drug [59]. Herein, we have investigated both unbound and total concentrations of cCPPs.

We first assessed the PK parameters of SFTI-1 and kalata B1 in a rat model, sampling blood peptide concentrations in male Sprague Dawley rats after a 10 min intravenous infusion of 0.5 mg/kg of individual peptides in saline (Figure 1a). SFTI-1 and kalata B1 exhibited plasma half-lives of 20 and 35 min, respectively (Table 2, Figure 1b). They exhibited similar systemic clearance with 1.82 mL/min/kg for SFTI-1 and 1.60 mL/min/kg for kalata B1 (Table 2). However, the volume of distribution differed more pronouncedly between the peptides, i.e., 191 mL/kg for SFTI-1 and 322 mL/kg for kalata B1, indicating that SFTI-1 is primarily distributed in body fluids with minimal tissue distribution (Table 2). SFTI-1 was mainly unbound in the plasma, with an unbound fraction (f_u,plasma_) of 0.94, while kalata B1 was more extensively bound, with an unbound fraction of 0.30.

We then evaluated the extent of BBB transport in the rat model under steady-state conditions after a 4 h constant-rate infusion of individual peptides (Figure 1a). Repeated measurements of total plasma concentrations confirmed the achievement of steady state. At the end of the infusion, the total plasma concentrations of SFTI-1 and kalata B1 were 400 ± 46.9 nM and 1096 ± 149 nM, respectively (Table 2). The total brain-to-plasma ratio, K_p,brain_, of SFTI-1 was 0.06, whilst that of kalata B1 was 0.23 (Table 2). However, it is widely accepted that the extent of BBB transport is most accurately characterized by the unbound drug brain partition coefficient, K_p,uu,brain_, i.e., unbound brain-to-plasma concentration ratio [59,60,61]. By means of the Combinatory Mapping Approach (CMA; Methods) we revealed a relatively good extent of BBB transport of SFTI-1 for being a peptide, with a mean K_p,uu,brain_ of 0.13 (13%); that of kalata B1 was only 0.005, i.e., 0.5% equilibrating across the BBB (Table 2, Figure 1c). These values indicate while SFTI-1 movement through the BBB is hindered to some extent, it has easier access to the brain ISF space than kalata B1, which is highly restricted from entering the brain.

We next performed a simulation exercise utilizing the obtained BBB transport rate (presented below) and extent parameters to predict the total and unbound plasma concentration profiles, as well as unbound brain ISF concentration profiles, of the peptides up to 20 h after a 4 h constant-rate intravenous infusion of SFTI-1 or kalata B1 (Supplementary Table S3 and Supplementary Figures S1 and S2). The simulation revealed that similar exposure of unbound peptide in plasma would lead to an over 26-fold higher exposure of the brain ISF to unbound SFTI-1 than to unbound kalata B1 (Supplementary Figure S2). This may imply that for achievement of similar unbound exposure to SFTI-1, kalata B1 needs to have much higher dose, which might be associated with increased risk of peripheral side effects.

### 3.2. Limited Permeability of Peptides in the Human In Vitro BBB Model

The rate of BBB transport, often reported as permeability rate, can be assessed in in vitro models of the BBB [48,62]. Here, we investigated it in a human in vitro BBB model utilizing CD34^+^ endothelial cells derived from hematopoietic stem cells in co-culture with bovine brain pericytes [48,63] (Figure 4a,b). After 6 days of co-culture, endothelial cells acquired BBB phenotype and were then named brain-like endothelial cells (BLECs). We checked the integrity of the endothelial cell layer using the paracellular marker sodium fluorescein. We did not observe any toxicity or loss of integrity upon the addition of the tested concentrations of the investigated cCPPs. The in vitro endothelial permeability (P_e_) of sodium fluorescein, measured in all experiments, was between 0.47 and 0.54 × 10^−3^ cm/min (Table S4), indicating a tight and functioning cell layer. We assessed the permeability of SFTI-1 at 250 nM and 500 nM. The average P_e_ value was 0.228 × 10^−3^ cm/min across both concentrations (Figure 4c). Because of the low transcellular transport and bioanalytical limit of detection of kalata B1, we assessed its permeability at 500, 1000, 2000, and 4000 nM. Although some disparity among values for the three independent inserts used at each concentration was observed for the permeability to kalata B1 (Figure 4c), these disparities were not attributed to a difference in the tightness of the BLECs (as indicated by permeability to sodium fluorescein in the same range for all inserts) nor by a difference between the concentrations tested, and the average Pe of kalata B1 was 0.230 × 10^−3^ cm/min across all concentrations (n = 12). We did not detect any differences between the concentrations tested, indicating that the permeability of cCPPs was not concentration-dependent within the studied range. Similarly, there were no differences in the apparent permeability, P_app_, between the cCPPs or concentrations tested (Figure 4c). This indicated that both cCPPs have very low permeability across the monolayer of BLECs.

Finally, we determined intracellular peptide concentrations in the endothelial cell layer by collecting and lysing the BLECs at the end of the trans-endothelial transport experiment. We did not detect SFTI-1 in the lysed endothelial cells, indicating that there is no accumulation of SFTI-1 in the cells. By contrast, kalata B1 was present within the cells, with 0.19% of the initial amount of kalata B1 in the lysed endothelial cells pre-exposed to 500 nM peptide; 0.09% in cells exposed to 1000 nM peptide; 0.23% in cells exposed to 2000 nM peptide; and 3.50% in cells exposed to 4000 nM peptide. This indicated that kalata B1 may have high binding and accumulation in investigated endothelial cells yet did not impact the P_e_ or P_app_ values.

### 3.3. Peptide-Specific Differences in Intracellular Uptake

Many drug targets are located within the cell; hence, it is important to know the intra-cellular brain distribution of peptides and their propensity to enter neural cells after crossing the BBB. cCPPs enter the cell via passive diffusion and/or direct translocation as well as by energy-dependent active uptake [14]. One way of investigating the extent of intracellular distribution is by estimating the extent of cellular barrier transport from the unbound intracellular-to-extracellular concentration ratio, K_p,uu,cell_, a parameter that is not confounded by non-specific binding of the compound to cell constituents [53]. Direct measurements of K_p,uu,cell_ are not yet feasible, but the parameter can be estimated by combining a brain slice assay, assessing the overall uptake by brain cells, and brain tissue binding, measuring the primarily intracellular non-specific binding to cell constituents (Figure 1d).

We first used the brain slice assay to investigate the intra-brain distribution of cCPPs (Figure 2a). The unbound volume of distribution in the brain, V_u,brain_ (Methods, Equation (3)), at 37 °C was 0.436 mL/g brain for SFTI-1 and 152 mL/g brain for kalata B1 (Figure 2b, Table 3). The intra-brain distribution of kalata B1 was thus ca. 350-fold higher than that of SFTI-1. This reflects dramatic differences in the properties of these peptides related to their intracellular uptake and non-specific binding to brain parenchymal cells. Using equilibrium dialysis, we then investigated the non-specific binding of the peptides to brain tissue homogenate by calculating the fraction of unbound cCPP in the brain, f_u,brain_ (Methods, Equation (7)). SFTI-1 showed almost no brain tissue-binding capacity, while 93% of kalata B1 was bound to brain tissue constituents (Table 3). The mean V_u,brain_ value of kalata B1 was much higher than what would be expected from the peptide brain tissue binding (considering that V_u,brain_ ≈ 1/f_u,brain_), indicating that the contribution of the so-called non-specific brain tissue binding to overall distribution was minimal compared to the active processes taking place at the cellular membrane barrier, preserved in brain slices. Hence, we next performed additional exploratory experiments to elucidate the potential distributional mechanisms previously not investigated for kalata B1.

To investigate the involvement of active processes in the overall intra-brain distribution of cCPPs, we performed the brain slice assay at 4 °C. The intra-brain distribution of SFTI-1 was almost unchanged at 4 °C, with the mean V_u,brain_ value of 0.56 mL/g brain, indicating a restricted entry into the brain parenchymal cells (Figure 2b). By contrast, we observed a significant drop in V_u,brain_ of kalata B1 compared with measurements at 37 °C, from 152 to 1.12 mL/g brain (*p* < 0.0001), accordingly, reflecting the involvement of active uptake processes and their abolishment at low temperature (Figure 2b). Such low V_u,brain_ indicates homogenous distribution between intra- and extracellular compartments [39,59]. To test tissue-specificity of kalata B1 uptake, we then incubated the peptide with lung slices. V_u,lung_ was 1.23 mL/g lung, similar to the value obtained with the brain slice at 4 °C (Table 3, Figure 2b). This suggests that the cellular uptake of kalata B1 is brain-specific.

We then investigated the potential role of endocytosis in the brain cell distribution of the cCPPs. To accomplish this, we pre-incubated the brain slices for 30 min with the dynamin inhibitor dynasore (50 µM) before adding individually respective cCPPs. This resulted in a moderate but significant (23%) reduction in the V_u,brain_ of kalata B1 (*p* < 0.01, Figure 2b) but not SFTI-1. We also analysed the role of pH partitioning in the intracellular peptide distribution, as it is one of the critical mechanisms governing drug distribution, by pre-incubating the brain slices for 30 min with monensin (50 nM), which uncouples the natural cell proton gradient. Monensin caused a moderate but significant (30%) reduction of V_u,brain_ for both peptides (*p* < 0.01, Figure 2b). The observations indicate that pH partitioning plays a moderate role in intra-brain distribution of both cCPPs (Figure 3a).

Next, to test the cell specificity of peptide distribution in the brain tissue, we evaluated cCPP uptake in cell cultures of neurons and astrocytes differentiated from human neuronal stem cells (Figure 2c, Table 3). We used total cell-to-unbound media concentration ratio, K_p,u,cell_ (mL/g total protein; Methods, Equation (11)), a measure of both the uptake and binding to the cells, that is essentially similar to the V_u,brain_ measurement. For SFTI-1, the K_p,u,cell_ value was 31.1 and 19.0 mL/g total protein in neurons and astrocytes, respectively. This was up to 5-fold lower than what we observed for kalata B1 (K_p,u,cell_ of 135 and 94.7 mL/g total protein in neurons and in astrocytes, respectively). Investigation of the fraction of unbound peptides in cell lysates revealed on average up to a 3-fold higher binding capacity for both peptides than that of brain tissue homogenate (Table 3). For kalata B1, the uptake by neurons and astrocytes, measured as K_p,u,cell_, was similar to the value of V_u,brain_ obtained in the brain slice assay, with a less than 2-fold difference between them. However, the neural cell measurements for SFTI-1 were strikingly different from the brain tissue data. Specifically, K_p,u,cell_ was on average 44-fold (astrocytes) and 70-fold (neurons) higher than the V_u,brain_ value determined in the brain slice assay. The fraction of unbound SFTI-1 in cell lysates with an f_u,cell_ value of 0.305 was on average 3-fold lower than f_u,brain_, indicating that non-specific binding of SFTI-1 to cell constituents in the cell culture was higher than that in the brain homogenate (Table 3). This indicates obvious dissimilarities in the distribution of cCPPs measured in organotypic brain slice assay and cell cultures.

Finally, for a direct comparison of the brain tissue slice data and neural cellular barrier uptake data, we used K_p,uu,cell_, which describes the unbound intracellular-to-extracellular ratio or the unbound cell-to-medium concentration ratio (Section 2, Equations (12) and (14)). This allowed us to compare cellular uptake in tissue slices and cell cultures. The analysis revealed peptide accumulation in neurons and astrocytes, with cultured cells exhibiting a higher capacity to accumulate SFTI-1 than the brain slices (Figure 3b,c, Table 3). SFTI-1 was predominantly restricted from crossing the cellular barrier in brain slices, with a K_p,uu,cell_ of 0.415. The extent of cellular barrier transport was stable and independent of experimental conditions, i.e., incubation at 4 °C, inhibition of endocytosis, or dissipation of the pH gradient, with mean values of 0.3–0.47 (Figure 3b). By contrast, kalata B1 extensively accumulated in the brain tissue slices with K_p,uu,cell_ of 9.7, i.e., a 9.7-fold higher unbound intracellular concentration than the ISF concentration (Figure 3b). K_p,uu,cell_ was significantly reduced, to varying degrees, depending on the experimental conditions. For instance, we observed a 138-fold reduction in K_p,uu,cell_, from 9.7 under 37 °C conditions to 0.0721 when the brain slices were incubated at 4 °C. This supported the involvement of active transport processes at the cellular membrane of neural cells. Overall, kalata B1 had a high propensity to accumulate in brain parenchymal cells, while SFTI-1 had restricted cellular barrier transport.

## 4. Discussion

We here present the first in vivo characterization of BBB transport and intra-brain distribution of the two prototypic cCPPs, SFTI-1 and kalata B1, to determine whether they are suitable candidates for scaffolds for CNS drug delivery and compare their in vivo behaviour with that suggested by in vitro cell-based studies. We show that SFTI-1 has a superior extent of BBB transport in vivo to kalata B1, in spite of both cCPPs having the same rate of transport measured as permeability in an in vitro human BBB model. In addition, we demonstrate restricted uptake of SFTI-1 and an active uptake of kalata B1 at the cellular barrier in brain parenchyma, confirming the cell-penetrating property of kalata B1 also for neural cells. Collectively, we provide neuroPK-based evidence for the potential of SFTI-1, but not kalata B1, as a scaffold for brain delivery of drugs that act on extracellular targets.

A major advantage of the current study is related to the methodology and experimental design focusing on the free-drug theory [37,56,59]. We evaluated the extent of BBB transport by means of K_p,uu,brain_ under steady-state conditions (Figure 1d). This parameter describes the net flux of cCPPs across the BBB, excluding its binding to plasma proteins and brain tissue, and thereby providing a clean measurement of BBB transport. We show that the extent of BBB transport of the two cCPPs is different: SFTI-1 is transported more efficiently than kalata B1. The K_p,uu,brain_ value of SFTI-1 (0.13) indicates that, with a caveat of an appropriate dosing schedule and depending on the cargo potency, SFTI-1 may reach effective levels of exposure in the brain ISF. Indeed, according to the performed simulation exercise, the concentration of unbound SFTI-1 in the brain ISF could reach up to 100 nM during a 4 h intravenous infusion of 0.5 mg/kg of SFTI-1 (Supplementary Figure S2). This concentration would possibly be enough for a cargo to have an affinity to a brain therapeutic target in a low nanomolar range. By contrast, the brain ISF concentration of unbound kalata B1 was 26-fold lower than that of SFTI-1, with an unbound plasma concentration similar to that of SFTI-1. This makes this cyclotide not suitable for CNS drug delivery, with only 0.5% equilibrating across the BBB. Of note, the validity of SFTI-1 for CNS drug delivery must be evaluated using specific cargos, as the cargo would likely affect its BBB transport properties. Remarkably, after the first characterization of SFTI-1 [25], there have been many investigations of this unique and small cyclic peptide with only one di-sulphide bond, proposing its application in drug delivery [26,27]. Yet, SFTI-1, to the best of our knowledge, has never been investigated for CNS drug delivery.

To better understand the BBB transport properties of the two cCPPs, i.e., the rate of transport across the BBB and accumulation in endothelial cells, we tested them in a human in vitro BBB model [48]. Both peptides exhibited similarly low and concentration-independent endothelial permeability of approximately 0.2 × 10^−3^ cm/min, which is ca. 2.5-fold lower than the permeability of sodium fluorescein, the marker of paracellular transport (Figure 2c). It is also lower than that of the cyclic peptide cyclosporine A (1.20 kDa; P_e_ = 0.72 × 10^−3^ cm/min) tested in the same model [63]. This suggests that both cCPPs have very low permeability across endothelial cells. The observation that SFTI-1 and kalata B1 showed similar permeability in the in vitro BBB model used in the current study highlights the notion that there is an important difference between permeability (a rate measurement) and extent measurements (such as K_p,uu,brain_), where the two peptides differed significantly at the BBB, making one a possible scaffold and the other not.

Of note, only kalata B1, not SFTI-1, accumulated in the endothelial cells in the in vitro BBB model. This may indicate potential differences in the mechanism of uptake into the endothelial cells and further sorting of the two peptides, although the exact mechanisms of BBB transport remain unclear. Previous studies of cell penetration by SFTI-1 and kalata B1 have been performed using epithelial cell models from peripheral tissues, such as HeLa cells and MCF-7 cells, and in synthetic membranes based on phosphatidylcholine [29,31,32,33]. Whilst these types of cells and membranes may contribute information about the cell-penetrating capabilities of these peptides, it is worth noting that they lack the barrier properties of the BBB.

We used K_p,uu,cell_ measurements to investigate the transport of unbound cCPPs across various cell membranes in organotypic tissue slices and in cell culture under steady-state conditions. In analogy with K_p,uu,brain_, the K_p,uu,cell_ parameter represents the extent of cellular barrier transport of unbound peptide that is not influenced by non-specific or specific binding (Figure 1d). Since the two parameters merely describe the transport properties, they are more informative and, hence, advantageous for characterizing the cell-penetrating ability of peptides than total drug partition coefficients, e.g., K_p,brain_. Furthermore, they can be used to compare cCPP transport capability in different systems, a comparison that has not been reported to date. Once inside the brain, kalata B1 showed a much higher intra-brain distribution than SFTI-1. Kalata B1 also exhibited a high degree of non-specific binding in the brain slice assay, something that could potentially be attributed to the hydrophobic patch on the surface of the peptide interacting with cell and membrane constituents. When we combined the values from the brain slice experiments and brain tissue binding to calculate the uptake into cells (K_p,uu,cell_), we observed a high level of accumulation of kalata B1 in the brain parenchymal cells. On the other hand, SFTI-1 was restricted from entering the cells and, to a large degree, remained in the ISF. Hence, SFTI-1 might not be a suitable scaffold for drugs if the target is located intracellularly. The high K_p,uu,cell_ value of kalata B1 (9.7) suggests that the cellular accumulation is driven by active processes, a conclusion also indicated by the dramatic, 136-fold, decrease in uptake at 4 °C compared to experiments at 37 °C, with passive diffusion of the peptide remaining as the only distribution pathway at 4 °C. As a caveat, when interpreting these results, one needs to bear in mind a possible decrease in membrane dynamics at 4 °C caused by reduced fluidity of the lipid bilayer. Of note, the high accumulation of kalata B1 in brain slices was not apparent in lung slices, suggesting tissue dependence of kalata B1 cellular accumulation (Figure 2). The V_u,brain_ value for kalata B1 obtained at 4 °C was remarkably similar to that obtained in the V_u,lung_ experiment, which further supports the notion of a tissue-specific uptake mechanism of kalata B1.

Furthermore, our exploratory mechanistic studies revealed that both monensin and dynasore reduced the accumulation of kalata B1 in brain slices, indicating that both pH-driven and dynamin-dependent processes may contribute to intra-cellular accumulation of the cyclotide. Since neither monensin nor dynasore completely abolished the intracellular accumulation of kalata B1, other mechanisms likely contribute to the observed phenomenon. It is possible that kalata B1 does not enter the cell via traditional endocytosis but rather by aggregating in the cell membrane, where it forms pores, allowing it to enter the cytoplasm, as suggested by Henriques et al., who investigated kalata B1 in a HeLa cell model [30]. The proposed mechanism depends on the lipid characteristics of the cell membrane, since kalata B1 is said to interact with the membrane phospholipids, in particular, phosphatidylethanolamines, to form pores. Remarkably, phosphatidylethanolamines are highly expressed in neuronal cells compared to other cell types, which, at least partially, may explain the observed differences in kalata B1 brain vs. lung accumulation. Overall, our findings are in line with the findings of Henriques et al. [30,33], indicating the contribution of both internalization into acidic cell compartments and endocytosis, partially via a dynamin-dependent mechanism, to the intracellular accumulation of kalata B1. The seemingly brain-specific uptake of kalata B1 by brain parenchymal cells and dependence on mechanism(s) other than dynamin-dependent processes share many similarities with the behaviour of botulinum neurotoxin [64]. The neurotoxin enters neurons, utilizing the classical clathrin-dependent endocytosis and other clathrin-independent pathways. This similarity might be useful in guiding further studies on the cellular accumulation of kalata B1-based peptides.

Of note, whilst SFTI-1 did not appear to accumulate in the brain parenchymal cells, its K_p,uu,cell_ values in the cell culture uptake studies in neurons and astrocytes were above unity, i.e., indicating active uptake as opposed to active efflux or restricted transport across the cell membrane observed in the brain slice assay. In addition, the unbound fraction of SFTI-1 in the cell lysate was lower than that in the plasma and tissue homogenate, indicating higher binding (Table 2 and Table 3). For kalata B1, both the binding to cell lysate and cellular uptake were in line with the observed extensive binding in the brain homogenate and high accumulation in brain parenchymal cells in the brain slice assay (Table 3, Figure 2 and Figure 3). However, comparison of the absolute K_p,uu,cell_ values for kalata B1 in tissue slice experiments and cell culture experiments revealed a discrepancy, i.e., a ca. 3-fold relative reduction of K_p,uu,cell_ in cell culture. Several factors could explain the observed tissue vs. cell culture differences related to the inherent dissimilarities between cell culture and tissue slices and the methodology used. Collectively, these observations indicate that in vitro cell culture does not fully reflect the properties of tissue in vivo or live, organotypic tissue slices. Hence, the observed peptide accumulations in neuron and astrocyte cultures need further study to fully understand their mechanisms of cellular uptake.

Finally, the observation that one peptide is highly able to penetrate parenchymal cells but at the same time is unable to cross the BBB, and that the other peptide is able to pass the BBB but shows limited cell penetration, reinforces the importance of studying the two properties separately. Our findings strengthen the well-known notion that the BBB is a highly specialized barrier that restricts the transport of compounds that are otherwise able to easily penetrate the cell membrane. This point is crucial for drug development initiatives studying cCPPs as scaffolds for CNS disorders, and it highlights the need to study peptide disposition in the brain for each cCPP in a quantitative manner. Such studies could preferably be performed using the CMA to test if a prospective treatment would result in achieving adequate target site concentration of the therapeutic agent [37].

## 5. Conclusions

In conclusion, using several relevant tissue and cell models, in vivo and in vitro comparisons, and designing the experiments with a focus on the assessment of free (unbound) cCPP and the achievement of equilibrium, we have, for the first time, described the systemic PK, BBB transport, and intracellular uptake of SFTI-1 and kalata B1. Integration of all findings of this study allows us to conclude that under an appropriate dosing regimen, SFTI-1 could reach high enough concentrations in the brain ISF so as to recommend it for further exploration as a scaffold for CNS drug delivery. Kalata B1 is not recommended for this purpose. The challenge moving forward is devising the delivery strategy of such a scaffold to ensure that the BBB-penetrating capabilities are retained and that the therapeutic moiety can be delivered in sufficient concentrations to the site of action.

## Figures and Tables

**Table 1:** Summary of characteristics of SFTI and kalata B1.

Name	Sunflower Trypsin Inhibitor-1 (SFTI-1)	Kalata B1 (KB1)
Structure	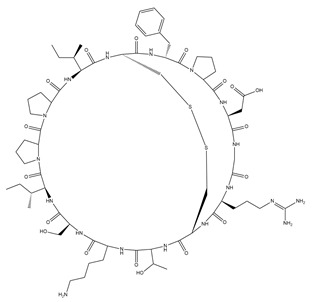	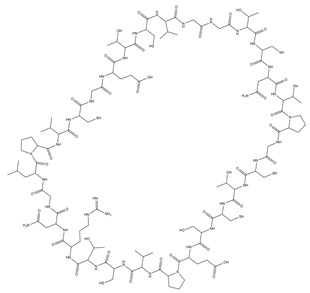
Amino acid sequence	GRCTKSIPPICFPD	GLPVCGETCVGGTCNTPGCTCSWPVCTRN
Molecular mass (Da)	1513	2892
Plant origin	*Helianthus annuus* (seeds), sunflower [20]	*Oldenlandia affinis* (leaves), Kalata-Kalata [21]
Number of disulphide bonds	1	3
Hydrophilicity	Amphiphilic	Amphiphilic with hydrophobic patch on surface [17]
Predicted net charge *	0	0
Biological activity	Trypsin inhibitor [20]	Uterotonic, plant defence peptide, nematocidal [22,23,24]

NB: the numbers refer to references; * estimates from PubChem.

**Table 2:** Systemic and brain distribution pharmacokinetic parameters (mean ± SD) of SFTI-1 and kalata B1.

Parameter	Unit	SFTI-1	Kalata B1
Systemic Parameters			
Area under the plasma drug concentration–time curve from time zero to infinity, AUC_0–∞_	µg × min/mL	77.3 ± 17.2	168 ± 38.1
Half-life, t_½_	min	20.3 ± 3.50	35.8 ± 3.52
Systemic clearance, CL	mL/min/kg	1.82 ± 0.480	1.60 ± 0.396
Apparent volume of distribution, V_d_	mL/kg	191 ± 34.4	322 ± 88.7
Fraction unbound in plasma, f_u,plasma_	(Unitless)	0.936 ± 0.038	0.299 ± 0.095
Brain distribution parameters			
Total brain-to-plasma concentration ratio, K_p,brain_	(Unitless)	0.0618 ± 0.0318	0.234 ± 0.083
Unbound brain-to-plasma concentration ratio, K_p,uu,brain_	(Unitless)	0.129 ± 0.0698	0.00479 ± 0.0023
Total steady-state plasma concentration, C_plasma,ss_	nM	400 ± 46.9	1096 ± 149

**Table 3:** Summary of pharmacokinetic parameters (mean ± SD) of tissue and cellular distribution, binding, and uptake of SFTI-1 and kalata B1.

Parameter	Unit	SFTI-1	Kalata B1
Brain			
Unbound volume of distribution in brain, V_u,brain_	mL/g brain	0.436 ± 0.064	152 ± 29.9
Fraction unbound in brain homogenate, f_u,brain_	(Unitless)	0.951 ± 0.115	0.0639 ± 0.0072
Unbound intracellular-to-extracellular (interstitial) concentration ratio, K_p,uu,cell_	(Unitless)	0.415 ± 0.079	9.73 ± 3.35
Lung			
Unbound volume of distribution in lung, V_u,lung_	mL/g brain	Not determined	1.22 ± 0.392
Fraction unbound in lung homogenate, f_u,lung_	(Unitless)	Not determined	0.0377 ± 0.0079
Unbound intracellular-to-extracellular concentration ratio, K_p,uu,cell_	(Unitless)	Not determined	0.195 ± 0.107
Neurons (N) and astrocytes (A)			
Fraction unbound in neuronal cell homogenate, f_u,N_	(Unitless)	0.347 ± 0.247	0.0153 ± 0.006
Fraction unbound in astrocyte homogenate, f_u,A_	(Unitless)	0.305 ± 0.039	0.0326 ± 0.004
Total neuronal cell-to-unbound medium concentration ratio, K_p,u,cell,N_	mL/g total protein	31.11 ± 12.46	135.35 ± 21.2
Total astrocyte cell-to-unbound medium concentration ratio, K_p,u,cell,A_	mL/g total protein	19.02 ± 4.34	94.73 ± 3.08
Unbound neuronal intracellular-to-extracellular concentration ratio, K_p,uu,cell,N_	(Unitless)	10.8 ± 12.00	2.08 ± 1.14
Unbound astrocyte intracellular-to-extracellular concentration ratio, *K_p,uu,cell,A_*	(Unitless)	5.8 ± 2.08	3.09 ± 0.53

## Data Availability

The datasets generated during and/or analysed during the current study are available from the corresponding author on reasonable request. The code used for simulation of time–concentration profiles for SFTI-1 and kalata B1 is presented in Supplementary Materials.

## Supplementary Materials

The following supporting information can be downloaded at: https://www.mdpi.com/article/10.3390/pharmaceutics15051507/s1. Table S1. Gradients used for cyclotide separation; Table S2. Settings of TQS-micro mass spectrometer for MRM quantification of SFTI-1 and kalata B1; Table S3. Pharmacokinetic parameters used in simulation exercise; Table S4. Permeability (Pe, 10^−3^ cm/min) to sodium fluorescein in in vitro BBB model in the presence or absence of SFTI-1 and kalata B1; Figure S1. Model and code used for simulation of time concentration profiles for SFTI-1 and kala-ta B1; Figure S2. Simulation of total plasma (red), unbound plasma (black), and unbound brain (blue) concentrations (nM) of SFTI-1 and kalata B1 up to 24 h after and during a 4-hour constant-rate intravenous infusion (IV inf.) of the respective peptides. Because most SFTI-1 is unbound in plasma (f_u,plasma_ is 0.936), the unbound and total plasma concentrations of SFTI-1 are overlapping. The initial total plasma concentrations were selected based on the achieved in vivo concentration in rats in the current study, i.e., 400 nM for SFTI-1 and 1000 nM for kalata B1. NB: for direct comparison between two cCPPs the concentrations are presented in nM; Figure S3. HPLC-UV chromatogram showing purity of SFTI-1, recorded at wavelength 215 nm. Gradient (in cyan) is the percentage of solvent B (0.045% TFA in ACN).
