# Supplementary material for: Differential Blood–Brain Barrier Transport and Cell Uptake of Cyclic Peptides In Vivo and In Vitro
Source: Pharmaceutics. 2023 May 16;15(5):1507. doi: 10.3390/pharmaceutics15051507 (PMC10222203; doi:10.3390/pharmaceutics15051507)
Supplement: Supplementary file 1 [file pharmaceutics-15-01507-s001.zip › pharmaceutics-2360298-supplementary.pdf]

pharmaceutics

Supplementary Material:

Differential Blood–Brain Barrier Transport and Cell Uptake of Cyclic Peptides In Vivo and In Vitro

Table S1. Gradients used for cyclotide separation.

Program (steps)	Time (min)	Flow rate (mL/min)	%MPA	%MPB
		**SFTI-1**		
**1**	0.00	0.300	100.0	0.0
**2**	0.50	0.300	100.0	0.0
**3**	6.00	0.300	60.0	40.0
**4**	6.50	0.300	100.0	0.0
		**Kalata B1**		
**1**	0.00	0.300	90.0	10.0
**2**	2.00	0.300	45.0	55.0
**3**	2.50	0.300	90.0	10.0

Mobile phase A (MPA) was 90 % MilliQ-water, 10 % acetonitrile, and 0.05 % formic acid. Mobile phase B (MPB) was 10 % MilliQ-water, 90 % acetonitrile, and 0.05 % formic acid. Separation was performed on a Peptide CSH C18 column (50×2.1 mm; particle size, 1.7 μm) (Waters, MA, USA).

Table S2. Settings of TQS-micro mass spectrometer for MRM quantification of SFTI-1 and kalata B1.

Parameter	Unit	SFTI-1	Kalata B1
**Capillary**	kV	3.40	1.70
**Cone**	V	40.00	10.00
**Source temperature**	°C	150	150
**Desolvation temperature**	°C	500	500
**Cone gas flow**	L/h	20	0
**Desolvation gas flow**	L/h	1100	1000
**Ion energy**	-	3.00	–1.10

Table S3. Pharmacokinetic parameters used in simulation exercise.

Parameter	Unit	SFTI-1	Kalata B1
	**Systemic parameters**		
**Systemic clearance, *CL***	mL/min/kg	1.82	1.6
**Systemic clearance, *CL***	L/h	0.0327	0.0288
**Systemic clearance unbound, *CLu***	L/h	0.0307	0.0086
**Apparent volume of distribution, V_d**	mL/kg	191	322
**Apparent volume of distribution, V_d**	L	0.0573	0.0966
**Apparent volume of distribution unbound, $V_{d,u}$**	L	0.054	0.029
**Fraction unbound in plasma, $f_{u,plasma}$**	(Unitless)	0.936	0.299
	**Brain distribution parameters**		
**P_{app}**	$\times 10^{-6}$ cm/s	2.62	3.19

**P_{app} rat**	×10^{-6} mL/s	471.6[a]	574.2[a]
**P_{app} rat**	mL/s	0.00047	0.000572
**CL_{in}**	L/h	0.00170	0.00207
**CL_{out}**	L/h	0.01317	0.43155
**Total brain-to-plasma concentration ratio, $K_{p,brain}$**	(Unitless)	0.0618±0.0318	0.234±0.083
**Unbound brain-to-plasma concentration ratio, $K_{p,uu,brain}$**	(Unitless)	0.129	0.00479
**Total steady-state plasma concentration, $C_{plasma,ss}$**	nmol/L	400	1096
**Total steady-state plasma concentration, $C_{plasma,ss}$**	mg/L	0.6052	3.169632
**Unbound steady-state plasma concentration, $C_{plasma,u,ss}$**	mg/L	0.5664672	0.947719968
**Unbound volume of distribution in brain, $V_{u,brain}$**	mL/g brain	0.436	152
**Unbound volume of distribution in brain, $V_{u,brain}$**	L	0.00078	0.2736
**Infusion rate**	mg/h	0.03816	0.03816
**Rat weight**	kg	0.3	0.3
**Brain weight**	g	1.8	1.8
**Surface area BBB***	cm^2	180	180
**Molecular mass**	Da	1513	2892

[a]Similar apparent permeability in human BBB cell culture model and rat endothelial cells was assumed.

* Hammarlund-Udenaes, M., Fridén, M., Syvänen, S. & Gupta, A. On the rate and extent of drug delivery to the brain. Pharm. Res. 25, 1737-1750 (2008).

Table S4. Permeability (Pe, 10^{-3} cm/min) to sodium fluorescein in in vitro BBB model in the presence or absence of SFTI-1 and kalata B1.

Treatment	Peptide concentration (nM)	Mean	SD
**Control**	0	0,46	0,12
**SFT1**	250	0,31	0,05
**SFT1**	500	0,32	0,02
**kalata B1**	500	0,50	0,10
**kalata B1**	1000	0,54	0,01
**kalata B1**	2000	0,47	0,07

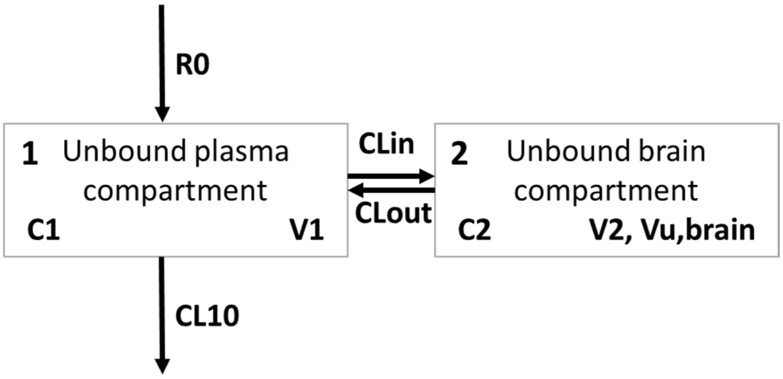

Figure S1. Model and code used for simulation of time concentration profiles for SFTI-1 and kalata B1.

1. SFTI-1

```
METHOD RK4
STARTTIME = 0
STOPTIME=24
DT = 0.02
d/dt(C1)=(R0-CL10*C1)/V1
d/dt(C2)=(CLin*C1-CLout*C2)/Vubrain
init C1 = 0.57; mg/L
init C2 = 0; mg/L
R0 = IF TIME <= INFSTOP THEN INFRATE ELSE 0
CL10 = 0.0307; L/h
CLin = 0.0016977; L/h
CLout = 0.01316; L/h
V1 = 0.054; L
Vubrain = 0.00078; L
INFRATE = 0.04; mg/h
INFSTOP = 4; h
```

2. Kalata B1

```
METHOD RK4
STARTTIME = 0
STOPTIME=24
DT = 0.02
d/dt(C1)=(R0-CL10*C1)/V1
d/dt(C2)=(CLin*C1-CLout*C2)/Vubrain
init C1 = 0.95; mg/L
init C2 = 0; mg/L
R0 = IF TIME <= INFSTOP THEN INFRATE ELSE 0
CL10 = 0.0086; L/h
CLin = 0.00206; L/h
CLout = 0.43; L/h
V1 = 0.0288 ; L
Vubrain = 0.274; L
INFRATE = 0.04; mg/h
INFSTOP = 4; h
```

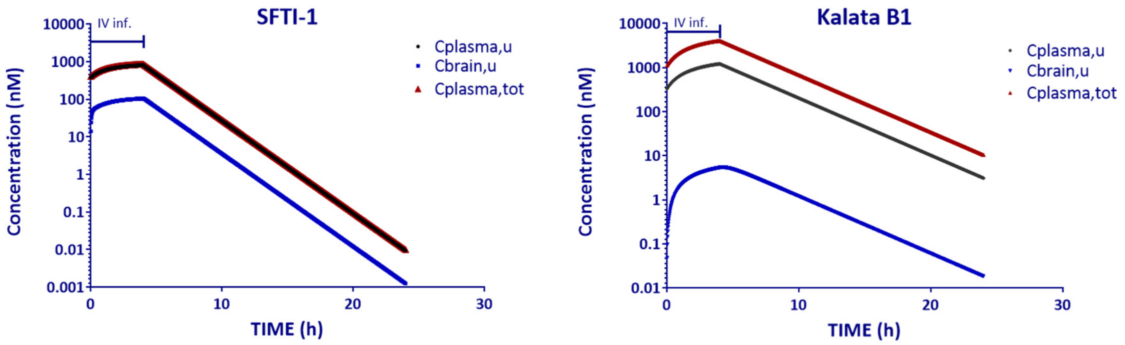

Figure S2. Simulation of total plasma (red), unbound plasma (black), and unbound brain (blue) concentrations (nM) of SFTI-1 and kalata B1 up to 24 h after and during a 4-hour constant-rate intravenous infusion (IV inf.) of the respective peptides. Because most SFTI-1 is unbound in plasma ($f_{u,plasma}$ is 0.936), the unbound and total plasma concentrations of SFTI-1 are overlapping. The initial total plasma concentrations were selected based on the achieved *in vivo* concentration in rats in the current study, i.e., 400 nM for SFTI-1 and 1000 nM for kalata B1. NB: for direct comparison between two cCPPs the concentrations are presented in nM.

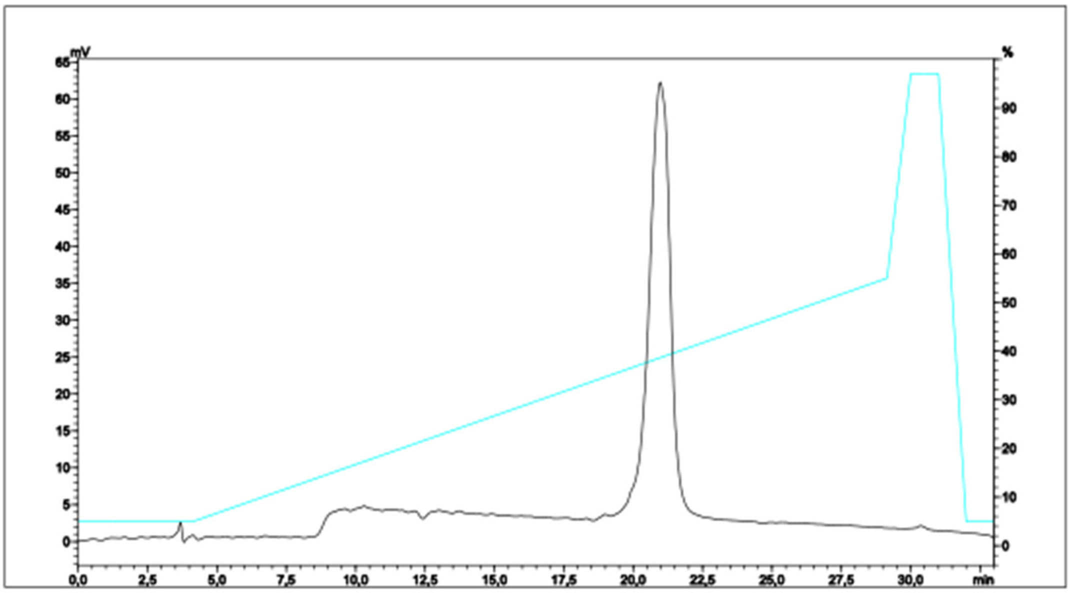

Figure S3. HPLC-UV chromatogram showing purity of SFTI-1, recorded at wavelength 215 nm. Gradient (in cyan) is the percentage of solvent B (0.045% TFA in ACN).
